# Emerging Roles of Non-Coding RNAs in the Feed Efficiency of Livestock Species

**DOI:** 10.3390/genes13020297

**Published:** 2022-02-03

**Authors:** Guoyu Hu, Duy Ngoc Do, Pourya Davoudi, Younes Miar

**Affiliations:** Department of Animal Science and Aquaculture, Dalhousie University, Truro, NS B2N 5E3, Canada; gy494270@dal.ca (G.H.); duy.do@dal.ca (D.N.D.); pourya.davoudi@dal.ca (P.D.)

**Keywords:** feed efficiency, miRNAs, lncRNAs, residual feed intake, food conversion ratio, pigs, chicken, sheep and cattle

## Abstract

A global population of already more than seven billion people has led to an increased demand for food and water, and especially the demand for meat. Moreover, the cost of feed used in animal production has also increased dramatically, which requires animal breeders to find alternatives to reduce feed consumption. Understanding the biology underlying feed efficiency (FE) allows for a better selection of feed-efficient animals. Non-coding RNAs (ncRNAs), especially micro RNAs (miRNAs) and long non-coding RNAs (lncRNAs), play important roles in the regulation of bio-logical processes and disease development. The functions of ncRNAs in the biology of FE have emerged as they participate in the regulation of many genes and pathways related to the major FE indicators, such as residual feed intake and feed conversion ratio. This review provides the state of the art studies related to the ncRNAs associated with FE in livestock species. The contribution of ncRNAs to FE in the liver, muscle, and adipose tissues were summarized. The research gap of the function of ncRNAs in key processes for improved FE, such as the nutrition, heat stress, and gut–brain axis, was examined. Finally, the potential uses of ncRNAs for the improvement of FE were discussed.

## 1. Introduction

The world’s population increased substantially in the last century and is expected to increase by 2 billion people in the next 30 years. While the need to increase animal production to meet global needs seems evident, the limited availability of resources is an important challenge in improving meat and milk production. Moreover, the cost of feed used in animal production has also increased dramatically, which requires animal breeders to find alternatives to reduce feed consumption while maintaining animal productivity. One available alternative to alleviate these problems is to emphasize feed efficiency in animal production. Genetic selection of feed-efficient animals via animal breeding approaches will provide a sustainable way of improving production while reducing feed intake. Thus, less pressure will be placed on the number of resources that are required for animal production. In addition, improving the feed efficiency of livestock has the potential to create a more environmentally friendly farming environment and provide better animal welfare for farmed animals.

In growing animals, ratio or residual (regression) traits can be used to describe feed efficiency [1]. Currently, many breeding companies use a feed conversion ratio (FCR) or feed:gain ratio as an indicator of feed efficiency. The FCR is computed as the total amount of feed intake divided into the total amount of gained weight. Koch et al. [2] introduced the concept of residual feed intake (RFI) and defined it as the difference between an animal’s actual feed intake and its predicted feed intake, the prediction usually being estimated based on energy requirements for maintenance, production, and body condition change. Therefore, a low RFI animal is more feed efficient than an animal with high RFI. Selection based on RFI has been proposed to improve feed efficiency because of its phenotypic independence on growth and maintenance requirements [3]. Some indirect measures of feed efficiency have also been proposed, such as relative growth rate (growth relative to instantaneous body size) [4] and the Kleiber ratio (ADG per unit metabolic BW) [5]. These two measures are rarely used in a breeding program, and the current breeding programs in livestock production mainly use either FCR or RFI as an indicator for improving feed efficiency.

Non-coding RNAs (ncRNAs) are transcribed RNA molecules that do not encode a protein [6], but play numerous regulatory roles in diverse biological processes, including epigenetic modification of DNA and regulation of transcriptional and post-transcriptional gene expression [7]. Amongst the most characterized ncRNAs are long non-coding RNAs (lncRNAs) and microRNAs (miRNAs). lncRNAs, which are at least 200 bases in length, can regulate gene expression through different mechanisms, including DNA methylation [8], histone modification [9], alteration of promoter activities by nucleosome repositioning [10], and epigenetic silencing and repression [11]. In the last few years, the increasing amount of evidence supports that lncRNAs are related to metabolic and immunological regulation and phenotypic variation of complex traits in domestic animals [12,13]. MicroRNAs (miRNAs) are small non-coding RNAs of 18 to 23 nucleotides and highly conserved between species [14]. Through the post-transcriptional regulation of gene expression technology, the miRNAs were found in all bodily fluids and tissues and most cell types [15] and were detected to be associated with different essential biological processes. They can regulate the gene expression through binding to target messenger RNA (mRNA), which ultimately leads to the degradation or inhibition of the targeted transcript. Recent studies have indicated that miRNAs have critical roles in regulating cellular and physiological processes, including neurogenesis, insulin secretion, cellular proliferation, differentiation, and apoptosis [16].

As the whole-genome sequencing of all livestock was completed, a tremendous success in the characterization of ncRNAs in farm animals was also observed. The studies associated with ncRNA in livestock have increased significantly in the last ten years (Table 1). More and more ncRNAs in livestock have been detected over the years. Not only the growth of the number of ncRNAs, but also more functions of ncRNAs in livestock have been described. Several reviews have examined the roles of ncRNAs in livestock phenotypes [6,12,13,17,18,19]. Given the importance of feed efficiency, it is essential to explore the miRNAs roles in the regulation of feed efficiency. This review provides the state-of-the-art studies related to the ncRNAs that underlie feeding efficiency in different livestock species. In this review, the contribution of ncRNAs to feed efficiency and related traits in the liver, muscle, and adipose tissues are summarized. Meanwhile, the research gap of the function of ncRNAs in related processes of feed efficiency, such as the nutrition, heat stress, and the gut–brain axis, are examined. Finally, the potential application of miRNAs in the improvement of feed efficiency are discussed. This review assists the elucidation of ncRNA mechanisms that could improve feed efficiency in livestock species.

## 2. The miRNAs Functions in Feed Efficiency

Pigs, cattle, sheep, and poultry are the most important farm animals for human society and provide the primary resource of milk, meat, egg, and wool. Some miRNAs in different tissues, including skeletal muscle, liver, and adipose tissues, have been discovered to have important roles in regulating feed efficiency in those important farm animals (Table 2). The detected feed-efficiency-related miRNAs help to better understand the molecular mechanisms and biological processes associated with feed efficiency in livestock.

### 2.1. The miRNA in Pig Feed Efficiency

**Figure 1:**
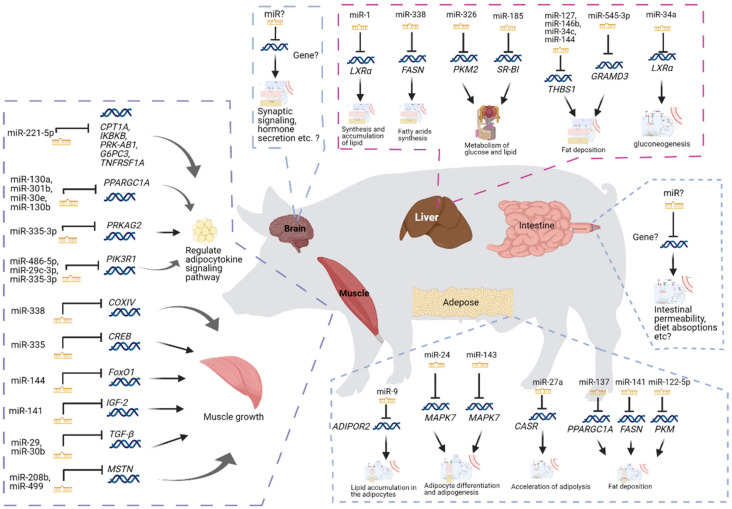
The miRNAs in pig feed efficiency. This figure was created with BioRender.com (2021).

Pig is an essential agricultural animal providing a cost-effective source of meat for human consumption. Over 40% of the world’s meat intake is pork [48]. More than 60% of the total costs of pig production is spent on feeding, and therefore, the critical approach to reduce costs in pig farming is improving feed efficiency. Feeding efficiency in pigs has been reported to be associated with many biological processes and pathways [20,49,50,51]. In pigs, miRNAs have been reported to play important roles in regulating feed efficiency in different tissues (Figure 1 and Table 2).

Skeletal muscle, which is the main place of carbohydrate and lipid metabolism, plays a significant role in utilizing and storing a large proportion of the energy obtained from the feed [52,53,54]. Therefore, the bioenergetic processes within the muscle can deeply influence feed efficiency. Some miRNAs detected in skeletal muscle correspond to two key pathways: one related to mitochondria and energy metabolism and the other related to skeletal muscle growth. Several miRNAs, including miR-338, miR-335, and miR-144, are related to mitochondria and energy metabolism [20,21]. More than 90% of cellular energy is produced by mitochondria through oxidative phosphorylation (OXPHOS) [55]. The miR-338 has been reported to inhibit COX-IV, one of the genes associated with OXPHOS and ATP synthesis and mitochondria transcriptional control, at mRNA and protein levels [56]. The PGC-1 protein is essential in mitochondrial biogenesis by activating cAMP response element binding protein (CREB) and nuclear respiratory factors. The miR-335, which was found to be up-regulated in low RFI pigs, has been reported to target the CREB [57]. The AMP-activated protein kinase (AMPK), which is a key regulator of cellular and whole-body energy balance, can increase mitochondrial proteins of oxidative metabolism, as well as promote the expression of Hexokinase II (HK2) through CREB in skeletal muscle [58,59,60]. The miR-144 has been reported to inhibit the phosphorylation of AMPK alpha, and therefore influence the level of energy metabolism in skeletal muscle [20,61]. The miR-221-5p has been reported to target *CPT1A*, *IKBKB*, *PRKAB1*, *G6PC3*, and *TNFRSF1A* genes to regulate the adipocytokine signaling pathway [22]. Several miRNAs, which include miR-208b, miR-499, miR-29, and miR-30b, were reported to be related to the growth and development of skeletal muscle [20]. Previous studies have reported that miR-29 and miR-30b are inhibitors of the transforming growth factor-beta (TGF-β) signaling pathway, which is considered the most potent negative regulator of skeletal muscle growth and development [62,63,64,65]. Both miR-208b and miR-499 have been reported to inhibit the gene expression associated with myostatin, which is an essential part of the TGF-β signaling pathway [66]. Meanwhile, miR-141 was reported to target the gene associated with insulin-like growth factor 2, which is an inhibitor of myogenesis in the myogenesis pathway, and therefore regulates the growth of skeletal muscle [20]. In addition, several miRNAs, including miR-130a, miR-301b, miR-30e, miR-130b, miR-335-3p, miR-486-5p, miR-29c-3p, and miR-335-3p, detected in skeletal muscle were reported to be involved in the adipocytokine signaling pathway [20].

The liver has been regarded as the central organ for systemic metabolism [67,68] and plays an essential role in feed efficiency, as it modulates the efficiency of converting energy obtained from macronutrients into muscle and/or adipose tissue [69]. Several miRNAs detected in pig’s liver were predicted to act as negative regulators of gene expression in fat formation. The miR-545-3p may regulate fat deposition by regulating the *GRAMD3* gene, which has been reported as a candidate gene for ectopic fat [23], because of the high negative correlation between *GRAMD3* and miR-545-3p [24]. The miR-338 was predicted to negatively regulate the genes associated with fatty acid synthase [24], which is a critical lipogenic enzyme and the rate-limiting step in de novo fatty acid synthesis [70]. The miR-127, miR-146b, miR-34c, and miR-144 have been predicted to inhibit the expression level of *THBS1* [24], which is important for the pathogenesis of insulin resistance and adipose tissue inflammation [71]. Several miRNAs detected in the liver of pig were predicted to be associated with the metabolism of glucose, lipid, and protein. For instance, miR-34a, miR-326, miR-1, and miR-185 were identified to participate in the metabolism of glucose and lipids [25]. The lower expression of miR-34a in pig liver can increase the expression of the *SIRT1* gene, which plays an important role in fat metabolism [72], and therefore increases gluconeogenesis [25]. The miR-1 was predicted to promote the synthesis and accumulation of lipids by increasing the expression of *LXRα* [25], that regulates the expression of genes associated with fatty acid synthesis, glucose metabolism, and sterol efflux [73].

The adipose tissue has been suspected of playing a vital role in feed efficiency, as it is the master regulator of systemic lipid storage and an active endocrine organ [74,75]. Several adipocytokines secreted by adipose tissue have been reported to communicate with skeletal muscle, liver and brain and influence various processes, including appetite, lipid, and glucose metabolism and energy homeostasis [74,76,77]. Several miRNAs detected in the adipose tissue were defined to be associated with adipogenesis. The overexpression of miR-9 in the adipose tissue may contribute to the lipid accumulation in the adipocytes [26]. The miR-24-3p has been reported to target the genes associated with adipocyte differentiation in MAPK7-signaling pathways and contribute to adipogenesis [27,78]. Several miRNAs detected in the adipose tissue were reported to be associated with adipocyte lipid metabolism. The miR-27a and miR-143 were reported to regulate the porcine adipocyte lipid metabolism [28]. The over-expression of miR-27a in adipose tissue could accelerate adipolysis to release more glycerol and free fatty acids. The over-expression of miR-143 could accumulate more triglycerides in the adipocytes and therefore promote adipogenesis [28]. Three miRNA, miR-137, miR-144, and miR-122-5p, were defined as the candidate key regulators of fat deposition [31].

In addition to liver, muscle, and adipose tissues, feed efficiency in pigs is also known to be involved in appetite and hormone regulation in the brain [79] as well as the microbiota [80,81] and nutrition digestion and absorption [82,83] in the gut tract. Although various genes have been reported to be involved in these processes, the information associated with how miRNAs can contribute to gene regulation in these tissues is still missing (Figure 1). Therefore, a comprehensive picture of feed efficiency regulations by miRNAs requires the exploration of their functions in the brain and gut.

Although many miRNAs have been reported to be related to feed efficiency in pigs, most studies focused on one tissue and the functions of some miRNAs. Comprehensive studies for miRNAs in multiple tissues for feed efficiency are still lacking. The discoveries of feed-efficiency-related miRNAs in pig’s liver, muscle, and adipose tissues would enhance our understanding of molecular mechanisms of the control of miRNAs in biological processes associated with feed efficiency.

### 2.2. The miRNA in Cattle Feed Efficiency

**Figure 2:**
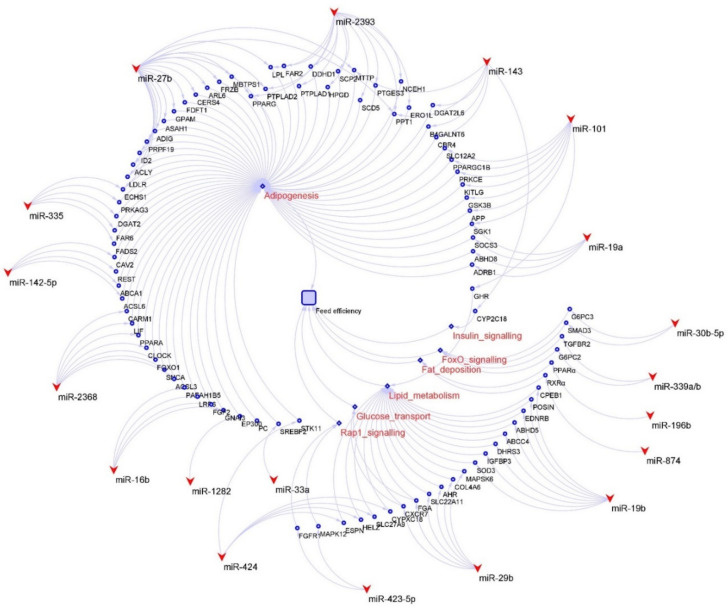
Candidate miRNAs and related genes and pathways for feed efficiency in cattle. The round shapes indicate genes, the diamond shapes show the pathways, and the V shapes indicate miRNA.

The unique ability of cattle to convert lignocellulosic biomass into valuable protein makes cattle one of the most crucial farm animals for human society. Approximately 45% of the global protein supply for humans is provided by meat and milk from cattle and bison [84]. Feed efficiency has some different aspects in the beef cattle and cows. Feed, which is one of the most important factors influencing the profitability of beef cattle farming, represents up to three-quarters of total beef production costs [85]. Compared with other livestock, such as pigs, chickens, and sheep, beef cattle have the lowest production efficiency [84]. Consequently, the cattle industry has shown great interest in improving the feed efficiency of beef production systems. In beef cattle, the miRNAs in the liver and skeletal muscle have been related to feeding efficiency [32,33,34,86,87]. In cows, in addition to the major tissues involved in feed efficiency, the milk production or lactation stages are also important since nutrition is used in the processing of producing milk [88,89]. In fact, the lactation stages could impact miRNAs’ expression and functions [90,91]. However, there is no research devoted to identifying miRNAs in both feed efficiency and lactation in cows. Several miRNAs have been related to regulating feed efficiency in different tissues (Table 2, Figure 2).

The liver, which is a central controller of metabolism and a significant driver of whole-animal oxygen consumption, plays a crucial role in the feed efficiency of cattle [32]. The main functions of miRNAs in the liver have been reported to regulate the energy metabolism and hepatic metabolism of nutrients, including lipids, carbohydrates, vitamins and minerals, and proteins and amino acids [92]. The feed efficiency is influenced by insulin and energy metabolism, and higher insulin and glucagon levels could reduce feed intake [50,93,94]. Several miRNAs in high RFI cattle have been reported to play important roles in metabolic homeostasis, including glucose and lipid metabolism. For example, miR-143, the most expressed miRNA in the bovine liver, was up-regulated in high RFI cattle [32] and reported to target insulin signaling and its regulation, and therefore inhibit the activation of insulin-stimulated AKT and the homeostasis of glucose homeostasis [95]. The miR-122-3p, which is linked to metabolic control and affects hepatic cholesterol and lipid metabolism [96], was also reported to be highly expressed in bovine liver and up-regulated in high RFI cattle [32]. The miR-29b, which has functions on regulating glucose transport in the liver, muscle, and adipose [97], was also reported to be up-regulated in the liver of high RFI cattle [32]. The miR-30b-5p and miR-339a/b were reported to target the genes associated with the FoxO signaling pathway, which regulates glucose metabolism and resistance to oxidative stress [98], and therefore contribute to higher feed efficiency in Nellore cattle [33]. In addition to those miRNAs, other cattle hepatic miRNA, such as miR-19b, miR-101, miR-106b, and miR-142-3p, were also reported to be up-regulated in high RFI cattle and correlated with lipid metabolism [32,38].

The metabolism of skeletal muscle greatly contributes to the variations in feed efficiency, as 30% of energy expenditure in cattle maintenance is used for turnover of body proteins, and approximately two-thirds of the whole-body protein turnover in mammals is associated with skeletal muscle and liver [33,34,99]. Therefore, the energy metabolism and growth of skeletal muscle have been suggested as a potential strategy for improving feed efficiency in bovines [33,34]. The miRNAs detected in the skeletal muscle of cattle have been reported to be related to the growth of skeletal muscle and energy metabolism in skeletal muscle in previous studies. The miR-423-5p in the skeletal muscle of cattle was found to be differentially expressed between low and high RFI beef cattle [33]. The author also reported that the miR-423-5p targets the genes associated with the Rap1 signaling pathway [33], which is associated with controlling mitogen-activated protein kinase activity [100] and regulating the storage of nutrients in the white adipose tissue and skeletal muscle [94]. The greater expression of miR-34a and miR-2899 was reported in the skeletal muscle of higher RFI cattle [34]. Both miRNAs were predicted to regulate the mRNA expression of heat shock protein beta 1 [34], which influences the degradation of muscle proteins and the rate of protein turnover in skeletal muscle [101]. The miR-148a-3p was found to be highly expressed in skeletal muscle and predicted to target the gene *KLF6*, which is very important for the development of skeletal muscles in bovines [35]. The skeletal-muscle-derived satellite cells (MDSCs) were reported to regulate postnatal skeletal muscle growth and regeneration. The growth of muscle tissue is highly correlated with the differentiation of MDSCs in cattle [102]. The expressions of 564 known and 53 novel miRNAs in hindlimb muscle were reported to be associated with the differentiation of MDSC in cattle [103]. The miR-224 and miR-130 were reported to impact on the differentiation of adipocytes [36,37].

The adipose tissue, the predominant anatomic site for lipogenesis in ruminants, modulates a large variety of processes related to feed intake, energy homeostasis, and whole-body physiology through its endocrinological activity [104]. The main functions of miRNAs in the adipose tissue have been reported to relate to adipose tissue metabolism and adipogenesis, which are highly associated with feed efficiency traits [39]. Several miRNAs including miR-16b, miR-19a, miR92a/b, miR-101, miR-103, miR106, miR-142-5p, miR-196a, miR-296, miR-2368, and miR-2454, were predicted to target genes associated with functions related to lipid metabolism and/or adipogenesis [38]. The miR-33a and miR-1281 in bovine adipose tissue were reported to, respectively, regulate the genes *SREBF2* and *EP300*, which are involved in lipid metabolism [39]. The miRNAs, including miR-143, miR-27, miR-335, and miR-378, were predicted to have important regulatory functions in adipose tissue and during adipogenesis [40]. MiRNAs were identified and predicted to regulate adipogenesis through their targets and related pathways [41]. Among them, miR-196b and miR-874 were predicted to influence the signal translation of the PPAR pathway, and therefore regulate fat deposition [41]. The miR-424 was reported to promote bovine adipogenesis through an unconventional post-transcriptional regulation of *STK11* gene [42]. In addition, 131 bovine miRNAs were predicted to upregulate the bovine adipocytes, and 119 bovine miRNAs were reported to downregulate bovine adipocytes [105].

While genomics has played an important role in improving feed efficiency in cattle [106,107,108], the biology underlying feed efficiency is still not completely understood. In beef cattle, protein turnover, tissue metabolism and stress, digestibility, heat increment, fermentation, and physical activity are the major processes contributing to the variation of residual feed intake [109], but very few studies focus on the roles of miRNAs in these processes. Similarly, some factors, such as feeding behaviors, physical activity, digestibility, and methane emissions, significantly contribute to the variation of feed efficiency in cows. The biological and physical aspects of these effects are not fully addressed. Therefore, it is worthwhile to investigate whether miRNAs are involved in the regulation of the process. Evidently, further studies are required to characterize the link between host miRNAs in regulating the rumen metabolites, the microbiome in divergent feed efficient cattle. Meanwhile, several miRNAs, such as miR-424 or miR-101, could impact the feed efficiency via regulating genes in different processes (Figure 2); therefore, these miRNAs might be prioritized for functional characterization. Nevertheless, the identified differentially expressed feed-efficiency-associated miRNAs in cattle not only help people to understand their potential molecular regulatory mechanisms relating to feed efficiency in cattle, but also provide potential candidate molecular targets for the selection of cattle with improved feed efficiency.

### 2.3. The miRNA in Sheep Feed Efficiency

**Figure 3:**
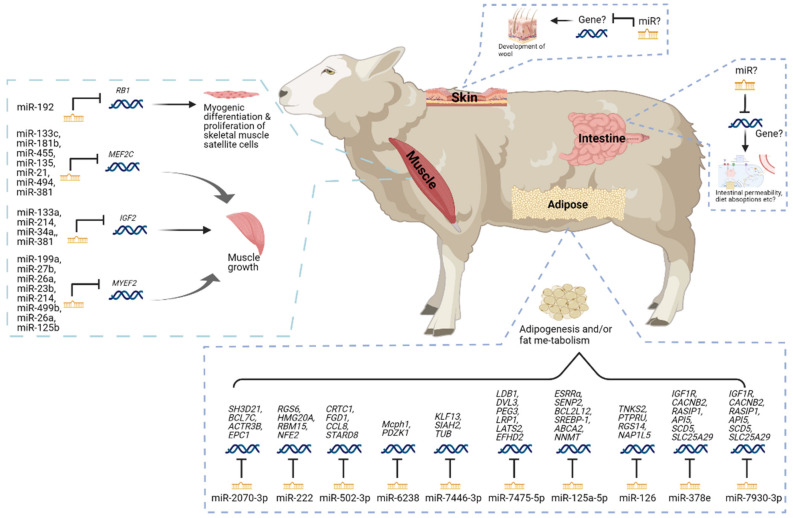
The miRNAs in sheep feed efficiency. This figure was created with BioRender.com (2021).

Sheep is one of the most crucial farm animals worldwide and provides high-quality meat and milk to human society. Increasing feed efficiency is important for the sheep farming industry to keep a stable output and improve the overall profitability from farming sheep because feed represents 65–70% of the total cost of sheep production systems [110,111,112]. The energy metabolism and growth of skeletal muscle play important roles in the feed efficiency of sheep, as skeletal muscle is a major tissue for energy utilization and maintenance of metabolic health as well as provides lean tissues for meat animals [113,114]. Several miRNAs in skeletal muscle and adipose tissue have been found to play important roles in regulating feed efficiency (Figure 3 and Table 2).

The miRNAs detected in the skeletal muscle of sheep have been related to the growth of skeletal muscle in previous studies. A total of 345 miRNAs, including 151 up-regulated and 94 down-regulated miRNAs, were differentially expressed in sheep’s skeletal muscle [43]. Among those miRNAs, some were predicted to be involved in the signaling transduction pathways associated with muscle development, such as the Wnt signaling pathway, hippo signaling pathway, and thyroid hormone signaling pathway [43]. Meanwhile, many of them were associated with regulating the growth of skeletal muscle. For example, miR-133c, miR-181b, miR-455, miR-135, miR-21, miR-494, and miR-381 were predicted to regulate the genes associated with myocyte enhancer factor 2 proteins [43], which are key transcriptional regulators of skeletal muscle development [115]. The miR-133a, miR-214, miR-34a and miR-381 were predicted to regulate the genes associated with insulin-like growth factor I [43], which plays a critical role in muscle regeneration [116]. The miR-199a, miR-27b, miR-26a, miR-23b, miR-214, miR-499b, miR-26a, and miR-125b were predicted to regulate the genes associated with myelin expression factor 2 [43], which contributes to the skeletal muscle differentiation [117]. A miRNA, miR-192, was reported to regulate the myogenic differentiation and proliferation of skeletal muscle sheep satellite cells [44], which are responsible for producing myoblasts in skeletal muscle [118], through regulating the repression of retinoblastoma 1, which is a known regulator of myogenesis [119]. Additionally, miR-1, miR-133b, miR-206, and miR-486 were also reported to be differential expressed in the skeletal muscle of sheep and predicted to correlate with the development of skeletal muscle [120].

The adipose tissue is not only one of the main sites for lipogenesis in ruminants, but also modulates and participates in a large variety of processes related to feed intake, energy homeostasis, and whole-body physiology through its endocrinological activity [104,121]. Therefore, the adipose tissue is highly correlated with feed efficiency in ruminants. Several miRNAs in sheep’s adipose tissue were reported to be associated with adipose tissue development and metabolism. Eight hundred fifteen miRNAs were found to be differentially expressed between fat-tailed (Kazakhstan) and thin-tailed (Tibetan) sheep breeds [45]. Among these miRNAs, several miRNAs, including miR-2070-3p, miR-222, miR-502-3p, miR-6238, miR-7446-3p, miR-7475-5p, miR-125a-5p, miR-126, miR-378e, and miR-7930-3p, were related to adipogenesis and/or fat metabolism in sheep. Meanwhile, a proportion of those 815 miRNAs were predicted to play roles in adiposity, adipocyte development and differentiation, and other metabolic disturbances in other species. For example, miR-378 was reported to target *MAPK1* and *PPAR* genes, which are associated with fat deposition and fatty acid metabolism, and promote bovine adipogenesis in white adipose tissue [122]; miR-103, miR-30, miR-27, and miR-138 have been reported to regulate adipogenesis [123,124,125,126,127,128]; miR-122, miR-370, and miR-378 have been reported to play important roles in lipid metabolism [129,130,131]; and miR-148a was reported to target *MAPKAPK5*, *MAPK3*, and *MAP2K2* genes and modulate fat deposition [132,133]. Additionally, 54 miRNAs were reported to be differentially expressed in 2 sheep breeds (Han and Dorset), including 35 down-regulated and 19 up-regulated miRNAs in the Han sheep. Among them, ten up-regulated miRNAs in the Han sheep were predicted to target 12 genes associated with enriching the lipid metabolic process [134].

One of the interesting aspects of feed efficiency in sheep is that the trait depends on the production needs as the feed efficiency for meat sheep might differ from the one for wool sheep. In addition to the muscle and adipose tissue (Figure 3), the skin might also be an important tissue for regulating miRNA in feed efficiency in sheep, as a part of energy might be used for the development of wool [135]. Meanwhile, the gastrointestinal tract has also been related to the feed efficiency in sheep [136,137], but little is known about the involvement of miRNAs in the gastrointestinal tract. In addition, compared to cattle or pigs, the miRNAs studies in feed-efficiency-related tissues, such as liver or digestive systems in sheep, are fewer. Further studies in these tissues will provide insight into the biological pathways and regulatory molecules related to the feed efficiency of sheep and form putative regulatory candidates for future research on feed efficiency traits in sheep.

### 2.4. The miRNA in Chicken Feed Efficiency

Increasing feed efficiency and breast yield is the major focus of the poultry industry to meet the growing consumer demand for white meat. In addition to particular success in genetic/genomic selection for larger and leaner chicken [138], genetic and genomic studies have significantly saved feeding costs and resources while increasing productivity and reducing greenhouse gas emissions [139,140]. In fact, current efforts to increase feed efficiency in broilers are primarily related to genetic selection. Genetic selection contributes about 85–90% to the increased feed efficiency in broiler, while feeding strategies and management are responsible for 10–15% of increased feed efficiency [141]. An important contributing factor for improving feed efficiency is the better understanding of genetics and biology underlying feed efficiency traits, thanks to many studies in gene mapping, transcriptomics, and other omics techniques [142,143,144]. To date, many QTLs linked to feed efficiency and its related traits have been deposited in the chicken QTL database (http://www.animalgenome.org/cgi-bin/QTLdb/GG/index (accessed on 8 July 2021). Although 882 precursor miRNAs, which generate 1232 mature miRNAs for *Gallus gallus* in the miRNA database miRBase (Release 22.1; www.miRbase.org (accessed on 10 August 2021), have been identified, limited numbers of miRNAs studies have been devoted to understanding the mechanism of feed efficiency [46,145] and related traits, such as growth [146] and skeletal muscle development [47,147,148,149], in chicken. In a genome-wide association study, Yuan et al. [46] identified three significant SNPs for feed efficiency traits located in the vicinity of the miR-15a. The authors performed enrichment analyses of the genes targeted by miR-15a and suggested this miRNA could play important roles in feed efficiency via controlling genes in the insulin signaling pathway, known for the regulation of appetite and feed intakes. Luo et al. [145] also identified a SNP (g.5678784A>T) in the miR-1596, which is important for RFI. No functional studies have been devoted to characterizing the roles of miRNAs in chicken feed efficiency. Using the systematic transcriptomics analyses of mRNAs and miRNA data, Li et al. [47] suggested that miR-142-5p can regulate FOXO3 in the regulation of the skeletal muscle growth in chickens. As mentioned above, the liver is a key tissue for feed efficiency and miRNAs, and Li et al. [150] identified 67 miRNAs higher at 20 weeks (pre-egg laying) and 13 miRNAs higher at 30 weeks (egg-laying) in this tissue of the Lushi hens. Hicks et al. [151] identified 40 differential expressed miRNAs when comparing the liver transcriptomics profiling of E18 and D3 chicken. Fat deposit is also an important process related to feed efficiency, and miRNAs have been indicated to play important roles in this process [152,153,154]. Some notable pathways related to the differentiation (in vitro) of primary chicken pre-adipocytes are MAPK signaling, insulin signaling, and fatty acid metabolism [154]. In fact, several miRNAs are suggested to play roles in liver function. For example, Ma et al. [155] reported miR-101-2-5p could target the *ApoB* gene in the liver of chicken (*Gallus Gallus*), and Tian et al. [156] showed miR-34a-5p targeted ACSL1 protein expression to increases hepatic triglycerides and total cholesterol levels in laying hens. In a recent study, Marchesi et al. [157] identified two miRNAs, miR-1730 and miR-1744, that were associated with the FCR trait in broilers. The authors also reported miRNAs related to the feed-efficiency-related traits, such as mir-1641, for feed intake or mir-1759 for body weight gain [158]. Notably, mir-1759 has been involved in triglyceride synthesis and adipocyte differentiation via regulating *LPIN1* [158].

Considering that feed accounts for approximately 70% of the total production cost in the poultry industry, it is important for the continued development of functional studies of miRNAs to improve feed efficiency in poultry. Several miRNAs (miR-1730, mir-1759, and miR-1744) could be used as biomarkers for the characterization of high and low feed efficiency animals. Given the fact that many mRNAs transcriptomic studies have been performed for feed efficiency in chicken [159,160,161,162], the integration of miRNAs sequencing with transcriptomics analyses might enhance the identification of miRNAs’ roles in feed efficiency in chicken.

## 3. Long Non-Coding RNA

Increasing feed efficiency is continuously gaining importance for ecological and economic reasons, as it has the potential to contribute to both increased productivity and reduced environmental impact in livestock production systems. In the past decade, more and more evidence has shown that lncRNAs play roles in regulating feed efficiency and related traits, including energy metabolism and the development of skeletal muscle in livestock species (Table 3).

In pigs, 811 lncRNAs were detected to be differentially expressed in the muscle and adipose tissue of piglets, which may result in differences in muscle and fat development [174]. Several lncRNAs were detected and predicted to regulate lipid metabolism and adipogenesis in pigs. Seventeen lncRNAs were reported to regulate eight genes associated with the PPAR signaling pathway [164], which is highly related to fatty acid and sterol metabolism as well as adipogenic differentiation [163]. The lncRNA, XLOC_014379, was reported to target enzyme SCD, which plays a critical role in transforming saturated fatty acid to endogenous oleic acid in food, regulating unsaturated fatty acid biosynthesis, and promoting lipid deposition [175], and thus regulating fatty acid metabolism [164]. Meanwhile, nine and eleven key lncRNAs were detected in the fatty acid metabolism and adipocyte differentiation networks, respectively [164]. In addition, the porcine PU.1 antisense lncRNA, which can form a sense–antisense RNA duplex with PU.1 mRNA to inhibit its translation, was reported to promote adipogenesis during the pre-adipocyte differentiation process [165].

In cattle, 126 lncRNA were reported to be associated with feed efficiency [166]. Among them, 71 detected lncRNAs (21 were identified in the adrenal gland, 8 in liver, 10 in muscle, 15 in the hypothalamus, and 17 in the pituitary gland) were identified as the key lncRNAs, which have the potential to regulate the expression of mRNA associated with feed efficiency in cattle [166]. Meanwhile, several of them were detected within the genomic regions of QTL for traits related to feed efficiency, feed intake, and fat deposition [166]. For example, TCONS_00119451 and TCONS_00119463 were reported to overlap seven QTLs, which include QTL:56461, QTL:20842, QTL:20843, QTL:20844, QTL:20845, QTL:20846, and QTL:20847, associated with RFI; TCONS_00032445, TCONS_00062811, and TCONS_00149966 were reported to overlap the QTLs related to dry matter intake; TCONS_00188391 and TCONS_00190543 were reported to coincide the QTLs associated with feed conversion ratio; and TCONS_00119451 and TCONS_00119463 were reported to overlap eleven QTLs related to fat deposition related traits [166]. Several other lncRNAs were predicted to be associated with energy metabolism in cattle. For example, MSTRG.4390 and MSTRG.5042 were reported to participate in the pathway enrichments for fatty acid β-oxidation and the TCA-cycle, respectively [167], which play important roles in mitochondrial function and energy metabolism for feed efficiency; MSTRG.4390 and MSTRG.5042 were also correlated with the expression of *PCK1* gene and *FBP1* gene [167], which occupy key roles in the biological pathway for energy balance in cattle through influencing gluconeogenesis [176]; and MSTRG.4802 was predicted to be involved in oxidative phosphorylation and mitochondrial dysfunction [167].

In sheep, some lncRNAs were found to be associated with feed efficiency and its related traits. Ten lncRNAs were identified as being differentially expressed between high and low RFI sheep [168]. Among them, LNC_000890 was predicted to regulate the metabolic efficiency of liver tissue metabolic efficiency and is co-expressed with the *ADRA2A* gene, which is significantly associated with feed efficiency [177], and therefore represents a crucial regulator for feed efficiency in sheep [168]. The lincRNA.16164 was predicted to target the *TSHZ1* gene [169], which is linked to body weight and lipid metabolism [178]. In addition, six lncRNAs were found to overlap with QTLs associated with tail fat deposition [169].

In chicken, there are some reports about some lncRNAs that regulate the development of skeletal muscle [170,171,179]. The lncRNA gga-lnc-0181 was found to be highly expressed in skeletal muscle and predicted to play a functional role in muscle development [170]. The lncRNA-Six1 was reported to regulate the *Six1* gene, which regulates the skeletal muscle development and transformation of muscle fiber types [180,181,182], thus promoting cell proliferation and being involved in muscle growth [171]. Several lncRNAs were detected and reported to be associated with the regulation of lipid metabolism. For example, the lncRNA, lnc_DHCR24, was repeated to be involved in lipid metabolism [172]. In addition, seven lncRNAs were found to be differentially expressed in the entire differentiation process of intramuscular preadipocytes, and therefore play an important role in the intramuscular preadipocytes [173].

Although the number of detected feed-efficiency-related lncRNAs is limited, the lncRNAs mentioned above in pig, cattle, sheep, and chicken demonstrate that lncRNAs also play important roles in regulating feed efficiency in livestock species. The poor accuracy of transcript detection caused by the limitations of high-throughput technologies and the low-level and extremely tissue-specific expression of lncRNAs is the major challenge associated with lncRNA analysis [12]. Only a small fraction of lncRNAs have been detected to be associated with feed efficiency in livestock species, and this knowledge gap highlights the need to explore the mechanisms of lncRNA in controlling the gene expressions related to feed efficiency.

## 4. Conclusions and Future Perspectives

In this paper, we have reviewed the role of ncRNAs in feed efficiency by emphasizing the state of the art of the current studies. Although a significant increase in the number of miRNAs related to feed efficiency in each livestock species was found, many research gaps should be filled in order to obtain a holistic picture of the ncRNAs for the traits. In cattle and pigs, many ncRNAs related to FE have already been identified, but information is still lacking for poultry. Since the major studies only focus on exploring or profiling ncRNAs related to the traits, the apparent need will be the functional characterization of their ncRNAs in vitro and in vivo. Moreover, many studies only look at one or some organs related to feed efficiency, and comprehensive approaches should be made. Although livers and muscles are major organs related to feed efficiency, the roles of GIT and brain in the regulation should not be ignored. For instance, miRNAs have been mentioned to play essential roles in the gut microbiota and brain signals in the diet response [183]. Therefore, it is expected that miRNAs could also mediate the regulation of feed efficiency-related genes in the gut–brain axis. The future of livestock farming will rely on precision nutrition [184,185]. Many ncRNAs have been linked to different specific diets and feeding formulas in livestock species. The question of using ncRNAs for a better animal diet, which could lead to improved feed efficiency, still needs to be answered. Heat stress is also important for feed efficiency, and several studies have indicated that miRNAs can play roles in the regulation of heat stress [186]. Developing some miRNAs as biomarkers for the selection of animals for both heat tolerance and improved tolerance should be prioritized in future research. Additionally, the ncRNAs have been shown to work together in the competitive endogenous networks [187]; therefore, the comprehensive understanding of ncRNA function needs to be put in the network perspectives. Consequently, future experiments should include several types of ncRNAs characterization at the same time.

From the livestock industry perspective, the most challenging question to be answered is how to use the knowledge of ncRNAs for improving feed efficiency. The development of ncRNA-based biomarkers is one of the options that can be considered. The combination of extracellular miRNAs with other phenotypic measurements will assess feed efficiency and related traits more accurately, allowing livestock producers to monitor feeding management and nutritional needs to optimize the use of feed. Another fascinating option is to use the genome-editing or RNA interfering tools to alter the miRNA expressions, consequently changing the downstream impact on the feed efficiency traits.

Finally, it is also worth mentioning the possible use of artificial intelligence, sensors, and big data to better understand the roles of ncRNAs on feed efficiency. Firstly, these methods and tools will help to better characterize the phenotype, which is the most important step before functional investigation. Secondly, deeper sequencing and better quality data will hopefully give a better capture of ncRNAs expression, especially the novel ncRNAs, and their link to the traits. Artificial intelligence, especially machine learning approaches, has been significantly involved in feed efficiency improvement. Their methods can also be used to accurately characterize ncRNAs functions in FE, such as the classification of ncRNAs involved in high and low feed efficient groups.

In conclusion, ncRNAs have a promising role in improving feed efficiency for major livestock species through regulating the expression of genes and pathways on FE-related organs. Although much needs to be performed to see the practical application of ncRNAs in reducing feed intake in the livestock industry, the rapid change in technologies and methods might help to shorten these research and application gaps.

## Figures and Tables

**Table 1:** Number of miRNAs and lncRNAs in livestock species.

Species	# QTLs for Feed Efficiency ^1^	# miRNAs Identified ^2^	# lncRNAs Identified ^3^
Pigs	350 (FCR) + 96 (RFI) = 446	408 (precursors) + 457 (mature) = 865	81,209
Cattle	121 (FCR) + 655 (RFI) = 776	1064 (precursors) + 1025 (mature) = 2089	15,071
Chicken	666 (FCR) + 140 (RFI) = 806	882 (precursors) + 1232 (mature) = 2114	13,753
Sheep	—	106 (precursors) + 153 (mature) = 259	1856
Goat	—	267 (precursors) + 436 (mature) = 703	4518
Duck	—	4 (precursors) + 8 (mature) = 12	1121

^1^ FCR and RFI are included as feed efficiency traits (Animal QTLdb); ^2^ miRbase database; ^3^ RNAcentral database.

**Table 2:** The miRNAs involved in the feed efficiency of different livestock species.

Species	Tissue	Dysregulated miRNAs	Targeted Genes	Related Pathways	References
Pig	Skeletal muscle	miR-338	*COXIV*	Oxidative phosphorylation, ATP synthesis, and mitochondria transcriptional control	[20]
		miR-335	*CREB*	Mitochondrial biogenesis/function and energy expenditure	[20]
		miR-144	*FOXO1*	Phosphorylation of AMP-activated protein kinase alpha	[20,21]
		miR-221-5p	*CPT1A*, *IKBKB*, *PRKAB1*, *G6PC3*, *TNFRSF1A*	Adipocytokine signaling pathway	[22]
		miR-29 and miR-30b	*TGF-β*	Myogenesis	[20]
		miR-141	*IGF-2*	Myogenesis	[20]
		miR-208b and miR-499	*MSTN*	Myogenesis	
		miR-130a, miR-301b, miR-30e, and miR-130b	*PPARGC1A*	Adipocytokine signaling pathway	[20]
		miR-335-3p	*PRKAG2*	Adipocytokine signaling pathway	[20]
		miR-486-5p, miR-29c-3p, and miR-335-3p	*PIK3R1*	Adipocytokine signaling pathway	[20]
	Liver	miR-545-3p	*GRAMD3*	Fat deposition	[23]
		miR-338	*FASN*	Fatty acid synthase	[24]
		miR-127, miR-146b, miR-34c, and miR-144	*THBS1*	Fat deposition	[24]
		miR-326	*PKM2*	Metabolism of glucose and lipid	[25]
		miR-185	*SCARB1*	Metabolism of glucose and lipid	[25]
		miR-34a	*SIRT1*	Gluconeogenesis	[25]
		miR-1	*LXRα*	Synthesis and accumulation of lipid	[25]
	Adipose tissue	miR-9	*ADIPOR2*	Lipid accumulation in the adipocytes	[26]
		miR-24	*MAPK7*	Adipocyte differentiation and adipogenesis	[27]
		miR-27a	*CASR*	Acceleration of adipolysis to release more glycerol and free fatty acids	[28,29]
		miR-143	*MAPK7*	Adipocyte differentiation and adipogenesis	[28,30]
		miR-137	*PPARGC1A*	Fat deposition	[31]
		miR-141	*FASN*	Fat deposition	[31]
		miR-122-5p	*PKM*	Fat deposition	[31]
Cattle	Liver	miR-143	*CYP2C18*	Insulin signaling and glucose homeostasis	[32]
		miR-122-3p	*COL3A1*	Hepatic cholesterol and lipid metabolism	[32]
		miR-29b	*CXCR7*, *FGA*	Glucose transport in the liver, muscle, and adipose tissue	[32]
		miR-30b-5p	*G6PC3*, *SMAD3*	FoxO signaling pathway	[33]
		miR-339a/b	*G6PC2*, *TGFBR2*	Target the genes associated with the FoxO signaling pathway	[33]
		miR-19b	*EDNRB*, *IGFBP3*, *POSIN*, *CPEB1*, *ABCC4*, *ABHD5*, *DHRS3*, *SOD3*, *NKIRAS1*	Lipid metabolism	[32]
		miR-101	*GHR*	Lipid metabolism	[32]
		miR-29b	*CXCR7*, *FGA*, *AHR*, *COL4A6*, *MAPSK6*, *SLC22A7*	Lipid metabolism	[32]
		miR-424	*HELZ*, *ESPN*, *CYPXC18*, *SLC27A6*	Lipid metabolism	[32]
	Skeletal muscle	miR-423-5p	*FGFR1*, *MAPK12*	Rap1 signaling pathway and storage of nutrients in the skeletal muscle	[33]
		miR-34a and miR-2899	*HSPB1*	Regulating myogenesis	[34]
		miR-148a-3p	*KLF6*	Proliferation and apoptosis of bovine muscle cells	[35]
		miR-224	*LPL*	Adipocyte differentiation	[36]
		miR-130	*PPARG*	Adipocyte differentiation	[37]
	Adipose tissue	miR-101	*SLC12A2*, *SGK1*, *PRKCE*, *PPARGC1B*, *KITLG*, *GSK3B*, *APP*	Lipid metabolism and/or adipogenesis	[38]
		miR-19a	*SOCS3*, *SGK1*, *ADRB1*, *ABHD5*	Lipid metabolism and/or adipogenesis	[38]
		miR-16b	*FGF2*, *GNAI3*, *LRP6*, *PAFAH1B2*	Lipid metabolism and/or adipogenesis	[38]
		miR-142-5p	*ABCA1*, *ACSL6*, *CAV2*, *REST*	Lipid metabolism and/or adipogenesis	[38]
		miR-2368	*ACSL3*, *CARM1*, *CLOCK*, *FOXO1*, *LIF*, *PPARA*, *SNCA*	Lipid metabolism and/or adipogenesis	[38]
		miR-33a	*SREBF2*	Lipid metabolism	[39]
		miR-1281	*EP300*	Lipid metabolism	[39]
		miR-143	*CBR4*, *MTTP*, *PC*, *DGAT2L6*, *PPT1*, *B4GALNT1*	Lipid metabolism and/or adipogenesis	[40]
		miR-27b	*ADIG*, *GPAM*, *ARL6*, *LPL*, *PTPLAD2*, *ECHS1*, *MTTP*, *FDFT1*, *CERS4*, *ACLY*, *PRPF19*, *PPT1*, *ASAH1*, *MBTPS1*, *LDLR*, *FRZB*, *ID2*, *PPARG*	Lipid metabolism and/or adipogenesis	[40]
		miR-335	*FADS2*, *PRKAG3*, *ECHS1*, *DGAT2*, *FAR2*	Lipid metabolism and/or adipogenesis	[40]
		miR-2393	*LPL*, *PTGES3*, *PTPLAD1*, *PTPLAD2*, *SCD5*, *HPGD*, *SCP2*, *FAR2*, *DDHD1*, *NCEH1*, *PPT1*, *PPARG*, *ERO1L*	Lipid metabolism and/or adipogenesis	[40]
		miR-27b	*ADIG*, *GPAM*, *ARL6*, *LPL*, *PTPLAD2*, *ECHS1*, *MTTP*, *FDFT1*, *CERS4*, *ACLY*, *PRPF19*, *PPT1*, *ASAH1*, *MBTPS1*, *LDLR*, *FRZB*, *ID2*, *PPARG*	Adipogenesis	[40]
		miR-196b and miR-874	*PPARα*, *RXRα*	Peroxisome proliferator-activated receptor alpha pathway	[41]
		miR-424	*STK11*	Adipogenesis	[42]
Sheep	Skeletal muscle	miR-133c, miR-181b, miR-455, miR-135, miR-21, miR-494, and miR-381	*MEF2C*	Skeletal muscle differentiation	[43]
		miR-133a, miR-214, miR-34a, and miR-381	*IGF2*	Skeletal muscle differentiation	[43]
		miR-199a, miR-27b, miR-26a, miR-23b, miR-214, miR-499b, miR-26a, and miR-125b	*MYEF2*	Myelin expression	[43]
		miR-192	*RB1*	Regulate the myogenic differentiation and proliferation of skeletal muscle sheep satellite cells	[44]
	Adipose tissue	miR-2070-3p	*SH3D21*, *BCL7C*, *ACTR3B*, *EPC1*	Adipogenesis and/or fat metabolism	[45]
		miR-222	*RGS6*, *HMG20A*, *RBM15*, *NFE2*	Adipogenesis and/or fat metabolism	[45]
		miR-502-3p	*CRTC1*, *FGD1*, *CCL8*, *STARD8*	Adipogenesis and/or fat metabolism	[45]
		miR-6238	*MCPH1*, *PDZK1*	Adipogenesis and/or fat metabolism	[45]
		miR-7446-3p	*KLF13*, *SIAH2*, *TUB*	Adipogenesis and/or fat metabolism	[45]
		miR-7475-5p	*LDB1*, *DVL3*, *PEG3*, *LRP1*, *LATS2*, *EFHD2*	Adipogenesis and/or fat metabolism	[45]
		miR-125a-5p	*ESRRα*, *SENP2*, *BCL2L12*, *SREBP-1*, *ABCA2*, *NNMT*	Adipogenesis and/or fat metabolism	[45]
		miR-126	*TNKS2*, *PTPRU*, *RGS14*, *NAP1L5*	Adipogenesis and/or fat metabolism	[45]
		miR-378e	*IGF1R*, *CACNB2*, *RASIP1*, *API5*, *SCD5*, *SLC25A29*	Adipogenesis and/or fat metabolism	[45]
		miR-7930-3p	*CABIN1*, *PCDHA2*, *PLXNA4*	Adipogenesis and/or fat metabolism	[45]
Chicken	Liver	miR-15a	*FOXO1*, *PDPK1,* *PRKAR2A*	Insulin-signaling pathway	[46]
	Skeletal muscle	miR-142-5p	*FOXO3*	Promoting growth-related gene expression	[47]

**Table 3:** Long non-coding RNAs in feed efficiency.

Species	Dysregulated miRNAs	Related Pathways	References
Pig	17 lncRNAs	Regulates eight genes associated with the PPAR signaling pathway	[163]
	XLOC_014379	Targets enzyme SCD, and thus regulate fatty acid metabolism	[164]
	9 lncRNAs	Participates in the fatty acid metabolism network	[164]
	11 lncRNAs	Participates in the adipocyte differentiation network	[164]
	PU.1 antisense lncRNA	Promotes adipogenesis during the pre-adipocyte differentiation process	[165]
Cattle	TCONS_00119451 and TCONS_00119463	Overlaps seven QTLs associated with residual feed intake	[166]
	TCONS_00032445, TCONS_00062811, TCONS_00149966	Overlaps the QTLs associated with dry matter intake	[166]
	TCONS_00188391, TCONS_00190543	Overlaps the QTLs associated with food conversion ratio	[166]
	TCONS_00119451, TCONS_00119463	Overlaps 11 QTLs associated with fat deposition related traits	[166]
	MSTRG.4390, MSTRG.5042	Participates in the pathway enrichments for fatty acid β-oxidation and the TCA-cycle	[167]
	MSTRG.4390, MSTRG.5042	Correlated with the expression of *PCK1* and *FBP1* genes	[167]
	MSTRG.4802	Involved in oxidative phosphorylation and mitochondrial dysfunction	[167]
Sheep	LNC_000890	Regulates the liver tissue metabolic efficiency co-expressed with the *ADRA2A* gene, and therefore represents a crucial regulator for feed efficiency in sheep	[168]
	lincRNA.16164	Targets the *TSHZ1* gene	[169]
	6 IncRNAs	Overlaps with QTLs associated with tail fat deposition	[169]
Chicken	lnc-0181	Highly expressed in skeletal muscle and predicted to play a functional role in muscle development	[170]
	lncRNA-Six1	Regulates the *Sine oculis homeobox 1* gene, and thus promotes cell proliferation and is involved in muscle growth	[171]
	lnc_DHCR24	Involved in lipid metabolism	[172]
	7 lncRNAs	Differentially expressed in the entire differentiation process of intramuscular preadipocytes, and therefore plays an important role in intramuscular preadipocytes	[173]

## Data Availability

Not applicable.
